# Acidovorax sacchari sp. nov., a pathogen causing red stripe of sugarcane in Japan

**DOI:** 10.1099/ijsem.0.006575

**Published:** 2025-02-05

**Authors:** Hiroyuki Sawada, Hirosuke Shinohara, Yusuke Takashima, Ken Naito, Mamoru Satou

**Affiliations:** 1 Research Center of Genetic Resources, National Agriculture and Food Research Organization (NARO), 2-1-2 Kannondai, Tsukuba, Ibaraki 305-8602, Japan; 2 Graduate School of Agriculture, Tokyo University of Agriculture, 1737 Funako, Atsugi, Kanagawa 243-0034, Japan

**Keywords:** *Acidovorax avenae*, leaf stripe symptom, red stripe disease, sugarcane, *Saccharum officinarum*, secretion system

## Abstract

Phytopathogenic bacteria (MAFF 311311^T^ and MAFF 311313) were isolated from sugarcane plants exhibiting leaf stripe symptoms associated with red stripe disease in Okinawa Prefecture, Japan. The strains were Gram-reaction-negative, aerobic, motile with one polar flagellum, rod-shaped and non-spore-forming. The genomic DNA G+C content was 69.0 mol%, and the major cellular fatty acids (>10 % of the total fatty acids) included summed feature 3 (C_16 : 1_* ω7c* and/or C_16 : 1_* ω6c*), C_16 : 0_ and summed feature 8 (C_18 : 1_* ω7c* and/or C_18 : 1_* ω6c*). Phylogenomic analyses using whole-genome sequences consistently placed these strains within the genus *Acidovorax*. However, their phylogenetic positions did not correspond to any known species within this genus. Comparative analyses, including average nucleotide identity and digital DNA–DNA hybridization with closely related species, yielded values below the thresholds for prokaryotic species delineation (95–96 and 70 %, respectively), with the highest values observed for *Acidovorax oryzae* ATCC 19882^T^ (93.98 and 54.3 %, respectively). Phenotypic characteristics, cellular fatty acid composition and a repertoire of secretion systems and their effectors can differentiate these strains from their closest relatives. The phenotypic, chemotaxonomic and genotypic data obtained in this study indicate that MAFF 311311^T^ and MAFF 311313 constitute a novel species within the genus *Acidovorax*, for which we propose the name *Acidovorax sacchari* sp. nov., with MAFF 311311^T^ (=ICMP 25276^T^) designated as the type strain.

## Introduction

The genus *Acidovorax*, a member of the family *Comamonadaceae* within the order *Burkholderiales*, was originally defined by Willems *et al*. [1] in 1990. As of the present composition, the genus encompasses 20 species with validly published and correct names, sourced from diverse environments such as soil, water, clinical materials and plants, according to the List of Prokaryotic names with Standing in Nomenclature (https://lpsn.dsmz.de) [2]. Notably, several species display plant pathogenic properties, including *Acidovorax avenae*, *Acidovorax citrulli*, *Acidovorax cattleyae*, *Acidovorax konjaci*, *Acidovorax anthurii*, *Acidovorax valerianellae *and *Acidovorax oryzae* [3,7]. *A. avenae*, formerly categorized as a subspecies of *A. avenae* (*A. avenae* subsp. *avenae*) [3], is recognized as the causal agent of bacterial blight and/or red/brown stripe diseases that affect various plants within the *Poaceae* (including barley, maize, oats, sorghum and sugarcane), *Theaceae* (tea) and *Strelitziaceae* (white bird of paradise) families. In contrast, *A. citrulli* (formerly *A. avenae* subsp. *citrulli*) infects plants belonging to the family *Cucurbitaceae*, whereas *A. cattleyae* (formerly *A. avenae* subsp. *cattleyae*) is associated with diseases in *Cattleya* and *Phalaenopsis* orchids. Furthermore, *A. konjaci*, *A. anthurii*, *A. valerianellae* and *A. oryzae* have been documented as pathogens in konjac (*Amorphophallus konjac*), anthurium (*Anthurium andreanum*), lambs’ lettuce (*Valerianella locusta*) and rice (*Oryza sativa*), respectively.

Sugarcane (*Saccharum* spp.), a principal source of sugar and ethanol, has substantial economic significance as a commercial crop and is cultivated globally in over 80 countries [5,6]. Red stripe disease, attributed to *A. avenae*, ranks among the three most important bacterial diseases of sugarcane and causes substantial yield losses in numerous sugarcane-growing countries [5,8]. This disease manifests with two distinct symptoms, leaf stripe and top rot, which may occur independently or concurrently under field conditions. *A. avenae*, the disease pathogen, exhibits considerable genetic diversity, with isolates from sugarcane in different regions clustering into distinct intraspecific groups based on various molecular typing methods [5,8].

In Japan, red stripe disease has also been observed in sugarcane (*Saccharum officinarum*), and *A. avenae* is presumed to be the causative pathogen [9], as observed in other affected countries [5,8]. Shinohara [10] conducted a survey of a disease outbreak in Okinawa Prefecture, Japan, in 2001, isolating the pathogen from leaf stripe symptoms (Fig. S1, available in the online Supplementary Material). Following confirmation of its pathogenicity to sugarcane, the isolated pathogen was identified as *A. avenae* through homology searches of the 16S rRNA gene sequences and phenotypic tests and subsequently deposited in the Genebank Project (https://www.gene.affrc.go.jp/databases-micro_search_en.php) of the National Agriculture and Food Research Organization (NARO), Japan [10]. However, a recent preliminary reassessment of the pathogen, archived in the NARO Genebank, suggested that it might be a new species of the genus *Acidovorax*.

This study aimed to elucidate the taxonomic affiliation of strains MAFF 311311^T^ and MAFF 311313, both isolated as pathogens of sugarcane red stripe disease that occurred in Okinawa in 2001 and deposited in the NARO Genebank by Shinohara [10]. Employing a polyphasic approach, this study meticulously examined and compared the characteristics of MAFF 311311^T^ and MAFF 311313 with those of related species. These findings revealed that these strains belong to the genus *Acidovorax* and represent a novel species named *A. sacchari* sp. nov.

## Genome features

The genomes of MAFF 311311^T^ and MAFF 311313 were sequenced using Nanopore technology. The strains were cultured on standard methods agar (plate count agar) plates (Nissui) for 3 days at 28 °C, and the bacterial cells collected were used for DNA extraction. High-molecular-weight genomic DNA was extracted and purified using the NucleoBond HMW DNA kit (Macherey-Nagel) according to the manufacturer’s instructions. Purified DNA (400 ng) was then used for library preparation using the Rapid Barcoding Kit SQK-RBK114.24 (Oxford Nanopore Technologies). Long-read sequencing was performed using the PromethION platform with the R10.4.1 PromethION flow cell FLO-PRO114M (Oxford Nanopore Technologies). Using MinKNOW version 23.04.6 and Guppy version 6.5.7 (Oxford Nanopore Technologies), Nanopore sequencing signals were subjected to real-time base-calling with a high-accuracy model, demultiplexing, adapter trimming and low-quality filtering with the following thresholds: >1000 bases and >9 Phred quality scores. The demultiplexed sequencing signals (pod5_pass) were further subjected to base-calling with the super-accuracy model (dna_r10.4.1_e8.2_400bps_sup@v4.3.0) using dorado version 0.5.3 (https://github.com/nanoporetech/dorado). For quality control prior to assembly, poor-quality data were excluded from the obtained raw FASTQ reads (MAFF 311311^T^: 98 517 reads, 798 Mb, *N*_50_ of 14 184 bp; MAFF 311 313 : 82 125 reads, 593 Mb, *N*_50_ of 12 599 bp) using chopper version 0.7.0 [11] by retaining only the reads with a minimum length of 5 kb with >20 Phred quality scores and trimming 100 bases from both ends of the respective retained reads. The quality-filtered FASTQ reads of each strain (MAFF 311311^T^: 36 708 reads, 502 Mb, *N*_50_ of 16 922 bp, mean read quality 22.2; MAFF 311 313 : 27 719 reads, 361 Mb, *N*_50_ of 15 898 bp, mean read quality 22.2) were *de novo* assembled using Flye version 2.9.3 [12] with default parameters except for the additional parameters ‘--genome-size 5.5 m’ and ‘--asm-coverage 50’. Each assembly was further polished by Medaka version 1.11.3 (parameter: ‘-m r1041_e82_400bps_sup_v4.3.0’) (https://github.com/nanoporetech/medaka) using the quality-filtered FASTQ reads of each strain used for the assembly process. The quality of the assembled genome sequences was assessed using the CheckM [13].

As a result, a 5 5 83 543-bp circular chromosome, with 69.0 mol% DNA G+C content, was obtained for both MAFF 311311^T^ and MAFF 311313, with 90-fold and 64-fold coverage, respectively (Table S1). Their G+C content was within the range of 62–70 mol% reported for *Acidovorax* species [3]. The assembled genomes were then annotated using the DDBJ Fast Annotation and Submission Tool (DFAST) pipeline version 1.2.20 (https://dfast.ddbj.nig.ac.jp) [14] (Table S1). The genome sequencing data obtained for MAFF 311311^T^ and MAFF 311313 were deposited in DDBJ/ENA/GenBank under the accession numbers AP035783 and AP035784, respectively.

To taxonomically assess the relationship between MAFF 311311^T^ and MAFF 311313, digital DNA–DNA hybridization (dDDH) analysis was performed using their genome sequences. Formula 2 of the Genome-to-Genome Distance Calculator 3.0 (GGDC 3.0) (https://ggdc.dsmz.de/ggdc.php#) [15,16] was employed for this purpose. The outcome revealed a significant value of 100.0%, surpassing the species delineation threshold (70%) for prokaryotes [17], thus indicating their inclusion within the same species framework.

Furthermore, to gain preliminary insights into the taxonomic affiliation of MAFF 311311^T^/MAFF 311313 through genome analyses, the Type Strain Genome Server (TYGS) (https://tygs.dsmz.de) [18] and the Taxonomy Check tool integrated within DFAST [14] were used. After uploading the MAFF 311311^T^ genome sequence to TYGS, dDDH values were calculated against the type strain genomes to identify the closest match. Despite the highest dDDH value against *A. oryzae* ATCC 19882^T^ (54.3%) (Table S2), it was below the prokaryotic species delineation cutoff [17]. Similarly, utilizing the fast average nucleotide identity (FastANI) algorithm [19] in the Taxonomy Check, *A. oryzae* ATCC 19882^T^ emerged as the closest match (Table S3), yet its FastANI value (94.264%) also remained below the threshold [95–96% average nucleotide identity (ANI)] for prokaryotic species demarcation [17]. These findings suggest the potential existence of a novel species within the genus *Acidovorax*, represented by MAFF 311311^T^ and MAFF 311313.

## 16S rRNA gene analyses

For the comprehensive identification of bacterial species closely associated with MAFF 311311^T^ and MAFF 311313, a homology search utilizing their 16S rRNA gene sequences was initially conducted, following established procedures [20,21]. Their partial sequences (1460 bp) were obtained using direct sequencing of the PCR products, which revealed identical matches between the two sequences. Moreover, these sequences concurred with those of the 16S rRNA genes, which existed in triplicate in the genomes of MAFF 311311^T^ and MAFF 311313. Subsequently, using the EzBioCloud database (https://www.ezbiocloud.net/identify) [22], a homology search was performed using the MAFF 311311^T^ sequence as a query, leading to the identification of 33 known species exhibiting significant similarity to MAFF 311311^T^, and corresponding 16S rRNA gene sequences were collected from the EzBioCloud database.

Next, the 16S rRNA gene sequences of the species, displaying high dDDH/ANI values with MAFF 311311^T^ in the prior genome analyses performed using TYGS and DFAST (Tables S2 and S3), were procured from GenBank. Using the pairwise nucleotide sequence alignment tool (https://www.ezbiocloud.net/tools/pairAlign) [22] within EzBioCloud, the similarity values against the 16S rRNA gene sequence of MAFF 311311^T^ were computed.

Finally, by integrating the data derived from EzBioCloud, TYGS and DFAST while eliminating duplicates, a total of 35 known species with validly published and correct names were identified as closely related to MAFF 311311^T^/MAFF 311313 (Table S4). Notably, *A. oryzae* ATCC 19882^T^ showed the highest sequence similarity (100.00%) to MAFF 311311^T^.

To establish a preliminary phylogenetic framework for MAFF 311311^T^ and MAFF 311313, phylogenetic analyses based on 16S rRNA gene sequences were conducted using the methodologies outlined in our previous studies [20,21]. These analyses included MAFF 311311^T^/MAFF 311313, the 35 species identified as closely related (Table S4) and *Burkholderia cepacia* ATCC 25416^T^ (chosen as an outgroup based on the analysis result of Du *et al*. [23]). Utilizing mega 11 version 11.0.13 [24], the neighbour-joining, maximum likelihood and maximum parsimony methods were employed to analyse their 16S rRNA gene sequences. Tree reliability was assessed using the standard bootstrap method with 1000 replicates. In the resulting phylogenetic trees (Fig. S2), MAFF 311311^T^, MAFF 311313, *A. oryzae* and *A. avenae* were united to form a monophyletic clade with a bootstrap value of 99%, together with *A. citrulli* and *A. cattleyae*.

## Comparative genomics and phylogenomics

A comprehensive analysis of the whole-genome similarity between MAFF 311311^T^ and its closely related species was conducted using ANI and dDDH metrics. The ANI values were computed utilizing both the ANI algorithm using blast (ANIb) (http://enve-omics.ce.gatech.edu/g-matrix/index) [25] and the orthologous ANI algorithm using USEARCH (OrthoANIu) (https://www.ezbiocloud.net/tools/ani) [26]. The dDDH values were determined according to formula 2 of GGDC 3.0 (https://ggdc.dsmz.de/ggdc.php#) [15,16]. The genome sequence of MAFF 311311^T^ was used as a query. Of the 35 closely related species selected for comparison with MAFF 311311^T^/MAFF 311313 (Table S4), 31 (Table 1) were included in the analyses, excluding four species (*Giesbergeria giesbergeri*, *Giesbergeria kuznetsovii, Giesbergeria sinuosa* and *Simplicispira soli*) because of the unavailability of their genome sequences.

**Table 1.** Genomic relationship between *A. sacchari* sp. nov. strain MAFF 311311^T^ and the type strains of the closely related species The ANIb, OrthoANIu and dDDH values were calculated using the genome-based distance matrix calculator [25], ANI calculator [26] and genome-to-genome distance calculator 3.0 (formula 2) [15,16], respectively. The data shown here are presented in descending order of their ANIb values calculated against the MAFF 311311^T^ genome sequence.

Species	Strain	Accession number *	ANIb (%)	OrthoANIu (%)	dDDH (%)
***A. sacchari***	MAFF 311311^T^	AP035783.1	100.00	100.00	100.0
***A. sacchari***	MAFF 311313	AP035784.1	100.00	100.00	100.0
*A. oryzae*†	ATCC 19882^T^	JMKU00000000.1	93.75	93.98	54.3
*A. avenae*†	ATCC 19860^T^	NC_015138.1	93.35	93.57	52.6
*A. citrulli*†	DSM 17060^T^	FNEY00000000.1	93.20	93.22	50.9
*A.cattleyae*†	DSM 17101^T^	FNJL00000000.1	92.32	92.53	48.1
*A. anthurii*	CFPB 3232^T^	QLTA00000000.1	84.99	84.10	27.3
*A. konjaci*	DSM 7481^T^	FOMQ00000000.1	84.83	83.90	26.9
*A. wautersii*	DSM 27981^T^	FONX00000000.1	84.40	82.15	25.7
*A. monticola*	KACC 19171^T^	NZ_CP060790.1	82.53	80.56	23.7
*A. valerianellae*	DSM 16619^T^	FMZC00000000.1	82.20	79.92	23.4
*Diaphorobacter oryzae*	DSM 22780^T^	jgi:1 030 805	81.87	80.02	23.0
*Simplicispira lacusdiani*	CPCC 100842^T^	QXGR00000000.1	81.74	79.64	23.1
*Diaphorobacter nitroreducens*	DSM 15985^T^	RJVL00000000.1	81.41	79.50	22.5
*Comamonas granuli*	NBRC 101663^T^	BBJX00000000.1	81.37	79.30	22.7
*A. soli*	DSM 25157^T^	FNQJ00000000.1	81.04	78.76	22.6
*A. kalamii*	KNDSW-TSA6^T^	NOIG00000000.1	80.90	78.38	22.3
*A. lacteus*	JCM 31890^T^	BAABEX000000000.1	80.87	77.82	22.2
*A. carolinensis*	NA3^T^	NZ_CP021361.1	80.76	78.44	22.3
*A. caeni*	R-24608^T^	FPBX00000000.1	80.73	78.44	22.1
*A. delafieldii*	DSM 64^T^	VJWE00000000.1	80.70	78.23	22.1
*A. benzenivorans*	D2M1^T^	JAPCKI000000000.1	80.70	77.85	22.1
*A. radicis*	N35^T^	AFBG00000000.1	80.70	78.01	22.4
*A. facilis*	DSM 649^T^	JAMXAX000000000.1	80.65	77.97	22.2
*A. temperans*	DSM 7270^T^	VFPV00000000.1	80.56	77.92	22.2
*Comamonas antarctica*	16-35-5^T^	NZ_CP054840.1	80.53	77.88	22.1
*A. defluvii*	DSM 12644^T^	QLTU00000000.1	80.43	77.89	21.8
*Xenophilus aerolatus*	DSM 19424^T^	JASCNX000000000.1	80.26	76.85	21.9
*Xylophilus ampelinus*	CECT 7646^T^	QJTC00000000.1	80.16	77.16	21.8
*Pseudacidovorax intermedius*	DSM 21352^T^	QQAV00000000.1	79.98	76.70	21.5
*Comamonas aquatica*	NBRC 14918^T^	BBJR00000000.1	79.72	76.60	21.3
*Diaphorobacter aerolatus*	KACC 16536^T^	NZ_CP060783.1	79.68	76.31	21.4
*Giesbergeria anulus*	ATCC 35958^T^	FOGD00000000.1	78.08	74.01	20.4

**Fig. 1.**
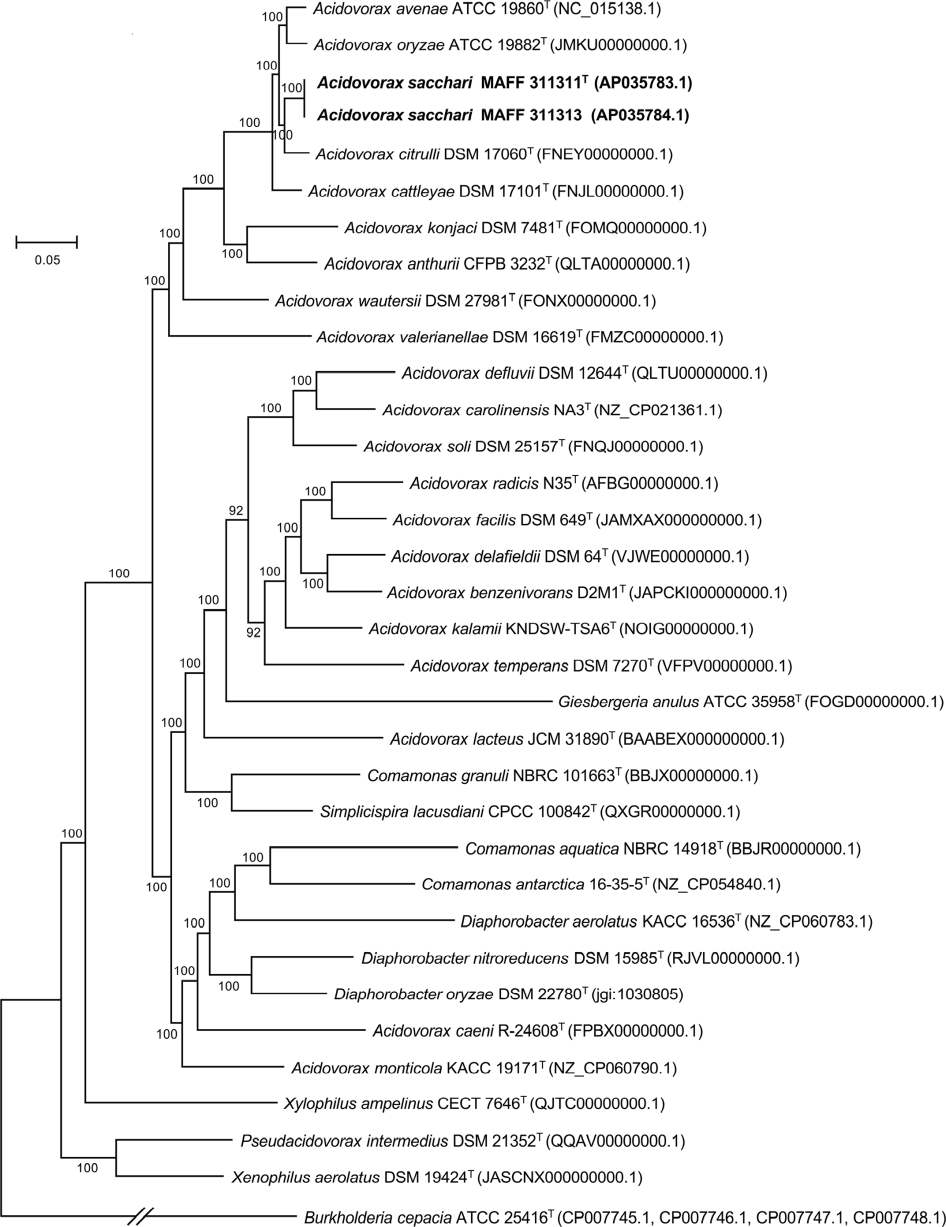
Phylogenomic tree was reconstructed based on the concatenated alignment of 246 core genes (total alignment length of 210 478 bp), showing the relationships between *A. sacchari* sp. nov. strains (boldface type) and the closely related species listed in Table 1. The concatenated alignment was generated using Roary [28] and Gblocks [30]. The maximum likelihood tree was inferred using RAxML-NG [31] with a general time-reversible substitution model and a gamma model of rate heterogeneity. *B. cepacia* ATCC 25416^T^ served as an outgroup. GenBank accession numbers are shown in parentheses. T, type strain of the species. Numbers at nodes indicate the standard bootstrap values from 100 replications.

*The whole-genome sequences used here are the same as those used in the phylogenomic analyses (Figs 1 and S3).

†The type strains of these four species were selected for comparison with MAFF 311311T/MAFF 311313 in the phenotypic and chemotaxonomic studies (Tables2  3) and functional genomics (Fig. S6, Table S6).

The ANI and dDDH values obtained for the type strains of the respective species are presented in Table 1 and are ordered by descending ANIb values against the MAFF 311311^T^ genome sequence. The ANIb values ranged from 78.08 (*Giesbergeria anulus* ATCC 35958^T^) to 93.75% (*A. oryzae* ATCC 19882^T^), while the OrthoANIu values ranged from 74.01 (*G. anulus* ATCC 35958^T^) to 93.98% (*A. oryzae* ATCC 19882^T^). Both ANI metrics fell below the threshold for prokaryotic species delineation [17]. Similarly, the dDDH values between MAFF 311311^T^ and its closely related species varied from 20.4 (*G. anulus* ATCC 35958^T^) to 54.3% (*A. oryzae* ATCC 19882^T^), all below the prokaryotic species delineation threshold [17].

To further elucidate the phylogenetic position of MAFF 311311^T^/MAFF 311313, phylogenomic analyses were performed using two distinct methodologies. First, a phylogenomic tree was constructed based on the concatenated alignment of core genes that constituted the core genome of MAFF 311311^T^/MAFF 311313, the 31 closely related species (Table 1) and *B. cepacia* ATCC 25416^T^ (outgroup). Genomic annotations were generated *de novo* using Prokka version 1.14.6 [27], and the annotated genes were compared across all input genomes using the Roary pan-genome analysis pipeline version 3.13.0 [28], with a 70% amino acid identity threshold. This process identified 246 core genes common to all the genomes analysed. The nucleotide sequences of these core genes were then concatenated, and multiple alignments were performed using MAFFT version 7.475 [29], implemented through Roary. To improve the alignment quality, poorly aligned positions and divergent regions were filtered out using Gblocks version 0.91b [30] with default parameters. From the resulting alignment, totalling 210 478 bp, a maximum likelihood tree was reconstructed using RAxML-NG version 1.2.2 [31] with a general time-reversible substitution model and a gamma model of rate heterogeneity. Tree reliability was assessed using the standard bootstrap method with 100 replicates. Additionally, phylogenomic analysis was performed using the TYGS web server [18]. The genome sequence dataset used in the above analysis was uploaded to TYGS and analysed using the genome blast distance phylogeny approach.

The topologies of the trees obtained using both methodologies were similar (Figs 1 and S3). MAFF 311311^T^ and MAFF 311313 formed a monophyletic and robust clade with high support values, along with *A. avenae*, *A. oryzae*, *A. cattleyae* and *A. citrulli*. However, the phylogenetic placement of MAFF 311311^T^/MAFF 311313 was distinct within this clade, not aligning closely with any other member.

## Physiology and chemotaxonomy

The phenotypic characteristics of MAFF 311311^T^ and MAFF 311313 were compared with those of the type strains of four *Acidovorax* species (*A. avenae*, *A. oryzae*, *A. cattleyae* and *A. citrulli*) that were most closely related to MAFF 311311^T^/MAFF 311313 in the phylogenomic analyses (Figs 1 and S3). The data for the latter four strains were retrieved from Bac*Dive* [32] for comparison. MAFF 311311^T^ and MAFF 311313 were cultured routinely at 28 °C on standard methods agar plates and tested for phenotypic characteristics, as described below. Cell size, morphology and flagellar insertion were determined using transmission electron microscopy, as described previously [20]. Growth at different temperatures (4, 6, 8, 10, 28, 37, 39, 41, 43, 45 and 47 °C) was evaluated using 1% peptone water without shaking. Gram reaction (Ryu non-staining KOH method), motility, fluorescent pigment production on King’s B medium (Eiken), oxidase activity, potato soft rot, tobacco hypersensitive reaction, yellow insoluble pigment production on nutrient agar and precipitate reaction in nutrient broth were examined according to the protocols described by Schaad *et al*. [33]. Poly-β-hydroxybutyrate accumulation was determined as described by Pierce and Schroth [34]. Catalase activity was determined using the method described by Lelliott *et al*. [35]. Additionally, biochemical and physiological characteristics were determined in duplicate using the API 20 NE kit (bioMérieux) according to the manufacturer’s instructions and as previously described [21].

MAFF 311311^T^ was rod-shaped with one polar flagellum (Fig. S4). The cell size (mean±sd) was 1.8±0.3 µm × 0.9±0.06 µm (*n*=28). Colonies of MAFF 311311^T^ and MAFF 311313 formed on standard methods agar plates were white/cream in colour, opaque/translucent, round with entire margins, slightly convex, smooth and glistening, with a diameter of ~1 mm, after culturing for 48 h at 28 °C (Fig. S5). The colonies reached diameters of ~2 mm after 3 days and 4 mm (maximum) after around 6 days of incubation. MAFF 311311^T^ and MAFF 311313 grew in the temperature range 10 to 41 °C, but not below 8 °C and not above 43 °C. These strains were Gram-reaction-negative and aerobic and exhibited positive responses for motility, oxidase activity, catalase activity, tobacco hypersensitive reaction and poly-β-hydroxybutyrate accumulation. However, they exhibited negative responses for fluorescent pigment production, potato soft rot, yellow insoluble pigment production on nutrient agar and precipitate reaction in nutrient broth. In API 20 NE tests, MAFF 311311^T^ and MAFF 311313 showed positive reactions for nitrate reduction, urease, assimilation of l-arabinose, d-mannitol, potassium gluconate, capric acid, adipic acid and malic acid and a weakly positive reaction for assimilation of trisodium citrate. However, negative reactions for indole production, glucose fermentation, arginine dihydrolase, aesculin hydrolysis, gelatin hydrolysis, β-galactosidase, assimilation of d-glucose, d-mannose, *N*-acetyl-d-glucosamine, maltose and phenylacetic acid were observed.

The differences in phenotypic characteristics between MAFF 311311^T^/MAFF 311313 and their closest relatives are summarized in Table 2. MAFF 311311^T^/MAFF 311313 could be distinguished from their closest relatives based on the following characteristics: positive for nitrate reduction, urease, assimilation of d-mannitol, capric acid and adipic acid; weakly positive for assimilation of trisodium citrate; and negative for arginine dihydrolase, aesculin hydrolysis, assimilation of d-glucose, d-mannose, *N*-acetyl-d-glucosamine, maltose and phenylacetic acid. The detailed phenotypic characteristics of MAFF 311311^T^/MAFF 311313 are provided in the species description section.

**Table 2.** Characteristics that differentiate *A. sacchari* sp. nov. strains from the type strains of the most closely related species Strains: 1–1, *A. sacchari* MAFF 311311^T^ (this study); 1–2, *A. sacchari* MAFF 311313 (this study); 2, *A. avenae* DSM 7227^T^ (data from Bac*Dive* [32]); 3, *A. oryzae* CCUG 15836^T^ [32]; 4, *A. cattleyae* DSM 17101^T^ [32]; 5, *A. citrulli* DSM 17060^T^ [32]. Characteristics have been scored as follows: +, positive reaction; −, negative reaction; w, weakly positive reaction; +/−, inconsistent results.

**Characteristics**	**1–1**	**1–2**	**2**	**3**	**4**	**5**
API 20 NE test*						
Nitrate reduction	+	+	+	+	−	+
Arginine dihydrolase	−	−	−	−	+/−	−
Urease	+	+	+	+	+	+/−
Aesculin hydrolysis	−	−	−	−	−	+/−
Assimilation of (API 20 NE)*						
d-Glucose	−	−	+	+	−	−
d-Mannose	−	−	−	+	−	−
d-Mannitol	+	+	+	+	+	−
*N*-Acetyl-d-glucosamine	−	−	−	+	−	−
Maltose	−	−	−	+	−	−
Capric acid	+	+	+	+	−	−
Adipic acid	+	+	+	+	+/−	+
Trisodium citrate	w	w	−	+	−	−
Phenylacetic acid	−	−	−	+	−	−
Isolation source	Sugarcane(*S. officinarum*)	Sugarcane(*S. officinarum*)	Corn(*Zea mays*)	Rice(*O. sativa*)	Unknown	Watermelon(*Citrullus lanatus*)
Genome size (Mb)†	5.58	5.58	5.48	5.53	5.61	4.85
DNA G+C content (mol%)†	69.0	69.0	68.8	68.8	68.6	68.9

*The results were scored after incubation for 4 days at 28 °C.

†These results were calculated from the respective whole-genome sequences.

For cellular fatty acid analysis, MAFF 311311^T^ was cultured on a trypticase soy agar plate for 48 h at 28 °C. Cellular fatty acids were then prepared and analysed according to our previous studies [20,21] using the Sherlock Microbial Identification System version 6.0 (midi) and the TSBA6 method. The major fatty acids (>10 % of the total fatty acids) in MAFF 311311^T^ (Table 3) were summed feature 3 (39.6 %), C_16 : 0_ (33.3%) and summed feature 8 (13.2 %). The composition ratios of the major fatty acid species were similar between MAFF 311311^T^ and its closest relatives [4,36]; however, differences were observed in the following minor fatty acid species (Table 3): C_15 : 0_, C_17 : 0_, C_17 : 0_ cyclo and C_15 : 1_.

**Table 3.** Cellular fatty acid composition (%) of *A. sacchari* sp. nov. strain MAFF 311311^T^ and the type strains of the most closely related species Strains: 1, *A. sacchari* MAFF 311311^T^ (this study); 2, *A. avenae* CFBP 2425^T^ (data from Gardan *et al*. [36]); 3, *A. oryzae* FC-143^T^ [4]; 4, *A. cattleyae* CFBP 2423^T^ [36]; 5, *A. citrulli* CFBP 10880^T^ [36]. tr, trace amount (<1 %); −, not detected. Fatty acids representing <1 % in all strains are not shown. The major fatty acids of each species (>10 % of the total fatty acids) are highlighted in bold.

Fatty acid	1	2	3	4	5
C_12 : 0_	2.7	2.6	2.1	2.3	2.3
C_14 : 0_	2.4	2.4	2.1	1.8	1.6
C_15 : 0_	–	tr	–	tr	6.7
C_16 : 0_	**33.3**	**32.2**	**32.3**	**35.2**	**33.7**
C_17 : 0_	tr	–	tr	tr	1.6
C_10 : 0_ 3OH	3.5	3.4	3.7	3.4	3.0
C_17 : 0_ cyclo	4.0	tr	tr	tr	–
C_15 : 1_	tr	–	–	–	2.6
Summed feature 3 *	**39.6**	**42.9**	**42.4**	**40.8**	**40.7**
Summed feature 8 †	**13.2**	**15.7**	**16.2**	**14.5**	7.6

*Summed feature 3: C_16 : 1_* ω7c/*C_16 : 1_* ω6c*.

†Summed feature 8: C_18 : 1_* ω7c/*C_18 : 1_* ω6c*.

## Functional genomics

The coding sequences in the MAFF 311311^T^ genome were annotated with eggNOG-mapper version 2.1.12 (http://eggnog-mapper.embl.de) [37] and with BlastKOALA version 3.0 from the Kyoto Encyclopedia of Genes and Genomes (KEGG) (https://www.kegg.jp/kegg/) [38]. Metabolic reconstruction was subsequently performed using KEGG Mapper version 5.1. Annotations from the eggNOG-mapper identified a high abundance of transcription-related genes (434 genes), followed by those potentially involved in amino acid transport and metabolism (424 genes), inorganic ion transport and metabolism (334 genes), energy production and conversion (314 genes) and signal transduction mechanisms (309 genes) (Table S5). KEGG annotation predicted that the MAFF 311311^T^ genome encodes 70 complete pathway modules organized into the following functional subcategories within the metabolism category: carbohydrate metabolism, energy metabolism, lipid metabolism, nucleotide metabolism, amino acid metabolism, glycan metabolism, metabolism of cofactors and vitamins, biosynthesis of terpenoids and polyketides and xenobiotics biodegradation. Notably, the genome was predicted to encode the complete pathway module for assimilatory nitrate reduction (KEGG Module: M00531) [39], aligning with the observed positive response of MAFF 311311^T^ to nitrate reduction (Table 2). Conversely, the genome was found to partially encode the arginine dihydrolase (deiminase) pathway for arginine degradation under oxygen-limiting conditions [40], which comprises three key enzymes: arginine deiminase (EC 3.5.3.6), ornithine carbamoyltransferase (EC 2.1.3.3) and carbamate kinase (EC 2.7.2.2). Among these, only the ornithine carbamoyltransferase gene was present, whereas the genes encoding the other two enzymes were absent, corroborating the experimental result (Table 2).

Previous studies have suggested that type II, type III and type VI secretion systems may play crucial roles in the pathogenicity of *Acidovorax* spp. in plants [41,45]. Therefore, it was investigated whether there are differences in the presence/absence of these secretion systems among MAFF 311311^T^ and its closely related species. Four phytopathogenic species (*A. avenae*, *A. oryzae*, *A. cattleyae* and *A. citrulli*), which the phylogenomic analyses have shown to be closely related to MAFF 311311^T^ (Figs 1 and S3), and two non-phytopathogenic species (*A. facilis* and *A. delafieldii*) were compared with MAFF 311311^T^. The total number of genes assigned by KEGG annotation to the core components of the type II (including Sec and Tat secretory pathways), type III (excluding the flagellar secretion system) and type VI secretion systems was calculated for each strain.

For the type II, Sec and Tat systems, relevant core genes were detected in all strains (Table S6), suggesting that these systems may play an essential role in the survival of these bacteria. In contrast, the number of genes assigned to the type III and type VI systems varied significantly among the strains. MAFF 311311^T^ and four phytopathogenic species (*A. avenae*, *A. oryzae*, *A. cattleyae* and *A. citrulli*) harboured type III and type VI secretion system genes, whereas no core genes associated with these systems were detected in the two non-phytopathogenic species (*A. facilis* and *A. delafieldii*) (Table S6). Based on comparative genomic analyses of *Acidovorax* strains, Siani *et al*. [45] proposed an evolutionary trajectory wherein ‘phytopathogenic *Acidovorax* strains’ emerged from free-living *Acidovorax* strains through the acquisition of type III and type VI secretion systems via horizontal transfer. The differences in the presence/absence of these secretion systems observed between phytopathogenic and non-phytopathogenic species in this study (Table S6) support the hypothesis of Siani *et al*. [45], suggesting the importance of these systems in the pathogenicity of ‘phytopathogenic *Acidovorax* strains’.

In *A. citrulli*, it has been suggested that intraspecific diversity in the repertoire of type III secreted effectors may contribute to variations in pathogenicity and host specificity, leading to group differentiation within the species [41,42, 44]. Therefore, identifying effectors specific to each phytopathogenic *Acidovorax* species could provide insights into the mechanisms underlying the differences in pathogenicity and host specificity among species. Towards this end, orthologous gene cluster comparisons among MAFF 311311^T^ and its closely related species were first performed using the OrthoVenn3 web server [46] with the following parameters (OrthoMCL algorithm, *E*-value 1×10^−2^, inflation value 1.50), aiming to identify genes specific to MAFF 311311^T^. Subsequently, these specific genes were analysed using the Bastion3 and Bastion6 web servers [47,48] to detect genes whose products might function as effectors secreted by type III or type VI secretion systems.

OrthoVenn3 analysis yielded 5057 orthologous gene clusters, of which 3444 clusters were common across all analysed genomes, while 5 clusters (comprising 10 genes) were unique to MAFF 311311^T^ (Fig. S6). Further analysis of these ten genes using the Bastion3 and Bastion6 web servers predicted five putative effectors that could be secreted by type III or type VI secretion systems (Tables S7 and S8). These putative effectors represent promising targets for future studies on the pathogenicity and host specificity of MAFF 311311^T^. Continued research in this area could elucidate the specific characteristics and evolutionary processes that lead to the pathogenic differentiation and speciation of MAFF 311311^T^. In addition, the putative effector genes listed in Tables S7 and S8 are specific for MAFF 311311^T^ and can therefore be used as markers in the development of identification and detection methods useful for epidemiological and ecological studies.

## Conclusions

Phylogenetic analyses of 16S rRNA gene sequences (Fig. S2), cellular fatty acid analysis (Table 3) and G+C content determination (Table 2), as well as preliminary genome analyses conducted via TYGS and DFAST (Tables S2 and S3), consistently indicated the affiliation of MAFF 311311^T^ and MAFF 311313 with the genus *Acidovorax*. Phylogenomic analyses using whole-genome sequences demonstrated that the phylogenetic positions of these strains did not correspond to those of any known species within the genus (Figs 1 and S3). Additionally, the results of the ANIb, OrthoANIu and dDDH analyses (Table 1) were consistent with the phylogenomic findings (Figs 1 and S3), confirming that MAFF 311311^T^/MAFF 311313 represent a novel species of the genus *Acidovorax*, for which we propose the name *A. sacchari* sp. nov., with MAFF 311311^T^ (=ICMP 25276^T^) as the type strain. Distinguishing features of *A. sacchari* sp. nov. from its closest relatives were observed in its phenotypic characteristics (Table 2), cellular fatty acid composition (Table 3) and repertoire of secretion systems and their effectors (Tables S6, S7 and S8).

Several issues regarding the pathogens of sugarcane red stripe disease remain unaddressed. First, determining whether all cases of the disease in Japan are caused by *A. sacchari* or if, similar to other affected countries [5,8], some cases are attributed to *A. avenae* is essential. Extensive epidemiological studies are needed to determine whether *A. sacchari* is distributed only in Japan or whether it is also involved in disease outbreaks outside Japan. In addition, detailed and multifaceted comparisons are necessary to determine whether there are differences in the pathogenicity, virulence and/or ecology between *A. sacchari* and *A. avenae* affecting sugarcane. We hope that the information obtained in this study will lead to further research addressing these issues.

## Description of *Acidovorax sacchari* sp. nov.

*Acidovorax sacchari* (sac'cha.ri. N.L. gen. n. *sacchari*, of the sugarcane genus *Saccharum*).

Members of this species are Gram-reaction-negative, aerobic, motile with one polar flagellum, rod-shaped and non-spore-forming. The cell size (mean±sd) is 1.8±0.3 µm × 0.9±0.06 µm (*n*=28). The strains of the species show growth in the temperature range 10 to 41 °C, but not below 8 °C and not above 43 °C. On standard methods agar (plate count agar) plates, the colonies are white/cream in colour, opaque/translucent, round with entire margins, slightly convex, smooth and glistening, with a diameter of ~1 mm, after culturing for 48 h at 28 °C. The strains are positive for oxidase activity, catalase activity, tobacco hypersensitive reaction and poly-β-hydroxybutyrate accumulation and are negative for fluorescent pigment production on King’s B medium, potato soft rot, yellow insoluble pigment production on nutrient agar and precipitate reaction in nutrient broth.

In API 20 NE tests (4 days incubation at 28 °C), positive responses are observed for nitrate reduction, urease, assimilation of l-arabinose, d-mannitol, potassium gluconate, capric acid, adipic acid and malic acid, and a weakly positive response is observed for assimilation of trisodium citrate; however, negative responses are observed for indole production, glucose fermentation, arginine dihydrolase, aesculin hydrolysis, gelatin hydrolysis, β-galactosidase, assimilation of d-glucose, d-mannose, *N*-acetyl-d-glucosamine, maltose and phenylacetic acid.

The major fatty acids (>10 % of the total fatty acids) in MAFF 311311^T^ are summed feature 3 (C_16 : 1_* ω7c* and/or C_16 : 1_* ω6c*), C_16 : 0_ and summed feature 8 (C_18 : 1_* ω7c* and/or C_18 : 1_* ω6c*). The genomic DNA G+C content of MAFF 311311^T^ is 69.0 mol%, and its genomic size is ~5.58 Mb.

The type strain, MAFF 311311^T^ (=ICMP 25276^T^), was isolated from a lesion formed on the leaf of sugarcane (*S. officinarum*) affected by red stripe disease, which was sampled in Okinawa Prefecture, Japan, in 2001, and is pathogenic to sugarcane. MAFF 311313 (=ICMP 25277) is an additional strain of this species.

Genome sequencing data for MAFF 311311^T^ and MAFF 311313 have been deposited in DDBJ/ENA/GenBank under the accession numbers AP035783 and AP035784, respectively. The 16S rRNA gene sequences of MAFF 311311^T^ and MAFF 311313 are available under the accession numbers LC797516 and LC797517, respectively.

## supplementary material

10.1099/ijsem.0.006575 Uncited Supplementary Material 1.

## Abbreviations

ANI: average nucleotide identity
ANIb: average nucleotide identity algorithm using BLAST
dDDH: digital DNA–DNA hybridization
DFAST: DDBJ Fast Annotation and Submission Tool
GGDC: Genome-to-Genome Distance Calculator
KEGG: Kyoto Encyclopedia of Genes and Genomes
NARO: National Agriculture and Food Research Organization
OrthoANIu: orthologous ANI algorithm using USEARCH
TYGS: Type Strain Genome Server

## References

[1] Willems A, Falsen E, Pot B, Jantzen E, Hoste B (1990). *Acidovorax*, a new genus for *Pseudomonas facilis*, *Pseudomonas delafieldii*, E. Falsen (EF) group 13, EF group 16, and several clinical isolates, with the species *Acidovorax facilis* comb. nov., *Acidovorax delafieldii* comb. nov., and *Acidovorax temperans* sp. nov. Int J Syst Bacteriol.

[2] Parte AC, Sardà Carbasse J, Meier-Kolthoff JP, Reimer LC, Göker M (2020). List of Prokaryotic names with Standing in Nomenclature (LPSN) moves to the DSMZ. Int J Syst Evol Microbiol.

[3] Willems A, Gillis M, Brenner DJ, Krieg NR, Staley JT, Garrity GM (2005). Bergey’s Manual of Systematic Bacteriology.

[4] Schaad NW, Postnikova E, Sechler A, Claflin LE, Vidaver AK (2008). Reclassification of subspecies of *Acidovorax avenae* as *A. avenae* (Manns 1905) emend., *A. cattleyae* (Pavarino, 1911) comb. nov., *A. citrulli* (Schaad et al., 1978) comb. nov., and proposal of *A. oryzae* sp. nov. Syst Appl Microbiol.

[5] Li X ‐Y., Sun H ‐D., Rott PC, Wang J ‐D., Huang M ‐T. (2018). Molecular identification and prevalence of *Acidovorax avenae* subsp. *avenae* causing red stripe of sugarcane in China. Plant Pathol.

[6] Fontana PD, Tomasini N, Fontana CA, Di Pauli V, Cocconcelli PS (2019). MLST reveals a separate and novel clonal group for *Acidovorax avenae* strains causing red stripe in sugarcane from Argentina. Phytopathology.

[7] Zhao J-Y, Chen J, Hu Z-T, Li J, Fu H-Y (2023). Genetic and morphological variants of *Acidovorax avenae* subsp. avenae cause red stripe of sugarcane in China. Front Plant Sci.

[8] Bertani RP, Mielnichuk N, Chaves S, Yaryura PM, Joya CM (2024). Comparative study of virulence factors, pathogenicity and genetic diversity of *Acidovorax avenae* subsp. *avenae*, the causal agent of red stripe disease in sugarcane. Plant Pathol.

[9] The Phytopathological Society of Japan (2024). Common Names of Plant Diseases in Japan. https://www.ppsj.org/pdf/mokuroku/mokuroku202402.pdf.

[10] Shinohara H (2003). Annual Report on Exploration and Introduction of Microbial Genetic Resources.

[11] De Coster W, Rademakers R (2023). NanoPack2: population-scale evaluation of long-read sequencing data. Bioinformatics.

[12] Kolmogorov M, Yuan J, Lin Y, Pevzner PA (2019). Assembly of long, error-prone reads using repeat graphs. Nat Biotechnol.

[13] Parks DH, Imelfort M, Skennerton CT, Hugenholtz P, Tyson GW (2015). CheckM: assessing the quality of microbial genomes recovered from isolates, single cells, and metagenomes. Genome Res.

[14] Tanizawa Y, Fujisawa T, Nakamura Y (2018). DFAST: a flexible prokaryotic genome annotation pipeline for faster genome publication. Bioinformatics.

[15] Meier-Kolthoff JP, Auch AF, Klenk H-P, Göker M (2013). Genome sequence-based species delimitation with confidence intervals and improved distance functions. BMC Bioinformatics.

[16] Meier-Kolthoff JP, Carbasse JS, Peinado-Olarte RL, Göker M (2022). TYGS and LPSN: a database tandem for fast and reliable genome-based classification and nomenclature of prokaryotes. Nucleic Acids Res.

[17] Riesco R, Trujillo ME (2024). Update on the proposed minimal standards for the use of genome data for the taxonomy of prokaryotes. Int J Syst Evol Microbiol.

[18] Meier-Kolthoff JP, Göker M (2019). TYGS is an automated high-throughput platform for state-of-the-art genome-based taxonomy. Nat Commun.

[19] Jain C, Rodriguez-R LM, Phillippy AM, Konstantinidis KT, Aluru S (2018). High throughput ANI analysis of 90K prokaryotic genomes reveals clear species boundaries. Nat Commun.

[20] Sawada H, Fujikawa T, Nishiwaki Y, Horita H (2020). *Pseudomonas kitaguniensis* sp. nov., a pathogen causing bacterial rot of Welsh onion in Japan. Int J Syst Evol Microbiol.

[21] Sawada H, Fujikawa T, Horita H (2020). *Pseudomonas brassicae* sp. nov., a pathogen causing head rot of broccoli in Japan. Int J Syst Evol Microbiol.

[22] Yoon S-H, Ha S-M, Kwon S, Lim J, Kim Y (2017). Introducing EzBioCloud: a taxonomically united database of 16S rRNA gene sequences and whole-genome assemblies. Int J Syst Evol Microbiol.

[23] Du J, Liu Y, Zhu H (2022). Genome-based analyses of the genus *Acidovorax*: proposal of the two novel genera *Paracidovorax* gen. nov., *Paenacidovorax* gen. nov. and the reclassification of *Acidovorax antarcticus* as *Comamonas antarctica* comb. nov. and emended description of the genus *Acidovorax*. Arch Microbiol.

[24] Tamura K, Stecher G, Kumar S (2021). MEGA11: Molecular Evolutionary Genetics Analysis Version 11. Mol Biol Evol.

[25] Rodriguez-R LM, Konstantinidis KT (2016). The enveomics collection: a toolbox for specialized analyses of microbial genomes and metagenomes. PeerJ Preprints.

[26] Yoon S-H, Ha S-M, Lim J, Kwon S, Chun J (2017). A large-scale evaluation of algorithms to calculate average nucleotide identity. Antonie van Leeuwenhoek.

[27] Seemann T (2014). Prokka: rapid prokaryotic genome annotation. Bioinformatics.

[28] Page AJ, Cummins CA, Hunt M, Wong VK, Reuter S (2015). Roary: rapid large-scale prokaryote pan genome analysis. Bioinformatics.

[29] Katoh K, Misawa K, Kuma K, Miyata T (2002). MAFFT: a novel method for rapid multiple sequence alignment based on fast Fourier transform. Nucleic Acids Res.

[30] Castresana J (2000). Selection of conserved blocks from multiple alignments for their use in phylogenetic analysis. Mol Biol Evol.

[31] Kozlov AM, Darriba D, Flouri T, Morel B, Stamatakis A (2019). RAxML-NG: a fast, scalable and user-friendly tool for maximum likelihood phylogenetic inference. Bioinformatics.

[32] Reimer LC, Sardà Carbasse J, Koblitz J, Ebeling C, Podstawka A (2022). Bac*Dive* in 2022: the knowledge base for standardized bacterial and archaeal data. Nucleic Acids Res.

[33] Schaad NW, Jones JB, Chun W. (2001). Laboratory guide for identification of plant pathogenic bacteria.

[34] Pierce L, Schroth MN (1994). Detection of Pseudomonas colonies that accumulate poly-β-hydroxybutyrate on Nile blue medium. Plant Dis.

[35] Lelliott RA, Billing E, Hayward AC (1966). A determinative scheme for the fluorescent plant pathogenic pseudomonads. J Appl Bacteriol.

[36] Gardan L, Dauga C, Prior P, Gillis M, Saddler GS (2000). *Acidovorax anthurii* sp. nov., a new phytopathogenic bacterium which causes bacterial leaf-spot of anthurium. Int J Syst Evol Microbiol.

[37] Cantalapiedra CP, Hernández-Plaza A, Letunic I, Bork P, Huerta-Cepas J (2021). eggNOG-mapper v2: functional annotation, orthology assignments, and domain prediction at the metagenomic scale. Mol Biol Evol.

[38] Kanehisa M, Sato Y, Morishima K (2016). BlastKOALA and GhostKOALA: KEGG tools for functional characterization of genome and metagenome sequences. J Mol Biol.

[39] Moreno-Vivián C, Cabello P, Martínez-Luque M, Blasco R, Castillo F (1999). Prokaryotic nitrate reduction: molecular properties and functional distinction among bacterial nitrate reductases. J Bacteriol.

[40] Cunin R, Glansdorff N, Piérard A, Stalon V (1986). Biosynthesis and metabolism of arginine in bacteria. Microbiol Rev.

[41] Burdman S, Walcott R (2012). *Acidovorax citrulli*: generating basic and applied knowledge to tackle a global threat to the cucurbit industry. Mol Plant Pathol.

[42] Eckshtain-Levi N, Munitz T, Živanović M, Traore SM, Spröer C (2014). Comparative analysis of type III secreted effector genes reflects divergence of *Acidovorax citrulli* strains into three distinct lineages. Phytopathology.

[43] Zeng Q, Wang J, Bertels F, Giordano PR, Chilvers MI (2017). Recombination of virulence genes in divergent *Acidovorax avenae* strains that infect a common host. Mol Plant Microbe Interact.

[44] Jiménez-Guerrero I, Pérez-Montaño F, Da Silva GM, Wagner N, Shkedy D (2020). Show me your secret(ed) weapons: a multifaceted approach reveals a wide arsenal of type III-secreted effectors in the cucurbit pathogenic bacterium *Acidovorax citrulli* and novel effectors in the *Acidovorax* genus. Mol Plant Pathol.

[45] Siani R, Stabl G, Gutjahr C, Schloter M, Radl V (2021). *Acidovorax* pan-genome reveals specific functional traits for plant beneficial and pathogenic plant-associations. Microb Genom.

[46] Sun J, Lu F, Luo Y, Bie L, Xu L (2023). OrthoVenn3: an integrated platform for exploring and visualizing orthologous data across genomes. Nucleic Acids Res.

[47] Wang J, Li J, Yang B, Xie R, Marquez-Lago TT (2019). Bastion3: a two-layer ensemble predictor of type III secreted effectors. Bioinformatics.

[48] Wang J, Yang B, Leier A, Marquez-Lago TT, Hayashida M (2018). Bastion6: a bioinformatics approach for accurate prediction of type VI secreted effectors. Bioinformatics.

