# Supplementary material for: Acidovorax sacchari sp. nov., a pathogen causing red stripe of sugarcane in Japan
Source: Int J Syst Evol Microbiol. 2025 Feb 5;75(2):006575. doi: 10.1099/ijsem.0.006575 (PMC11797039; doi:10.1099/ijsem.0.006575)
Supplement: Uncited Supplementary Material 1. [file ijsem-75-06575-s001.pdf]

IJSEM supplementary materials

Acidovorax sacchari sp. nov., a pathogen causing red stripe of sugarcane in Japan

Hiroyuki Sawada[1*], Hirosuke Shinohara[2], Yusuke Takashima[1], Ken Naito[1], and Mamoru Satou[1]

Author affiliations:

[1] Research Center of Genetic Resources, National Agriculture and Food Research Organization (NARO), 2-1-2 Kannondai, Tsukuba, Ibaraki 305-8602, Japan

[2] Graduate School of Agriculture, Tokyo University of Agriculture, 1737 Funako, Atsugi, Kanagawa 243-0034, Japan

*** Correspondence:**

Hiroyuki Sawada, sawada@naro.affrc.go.jp

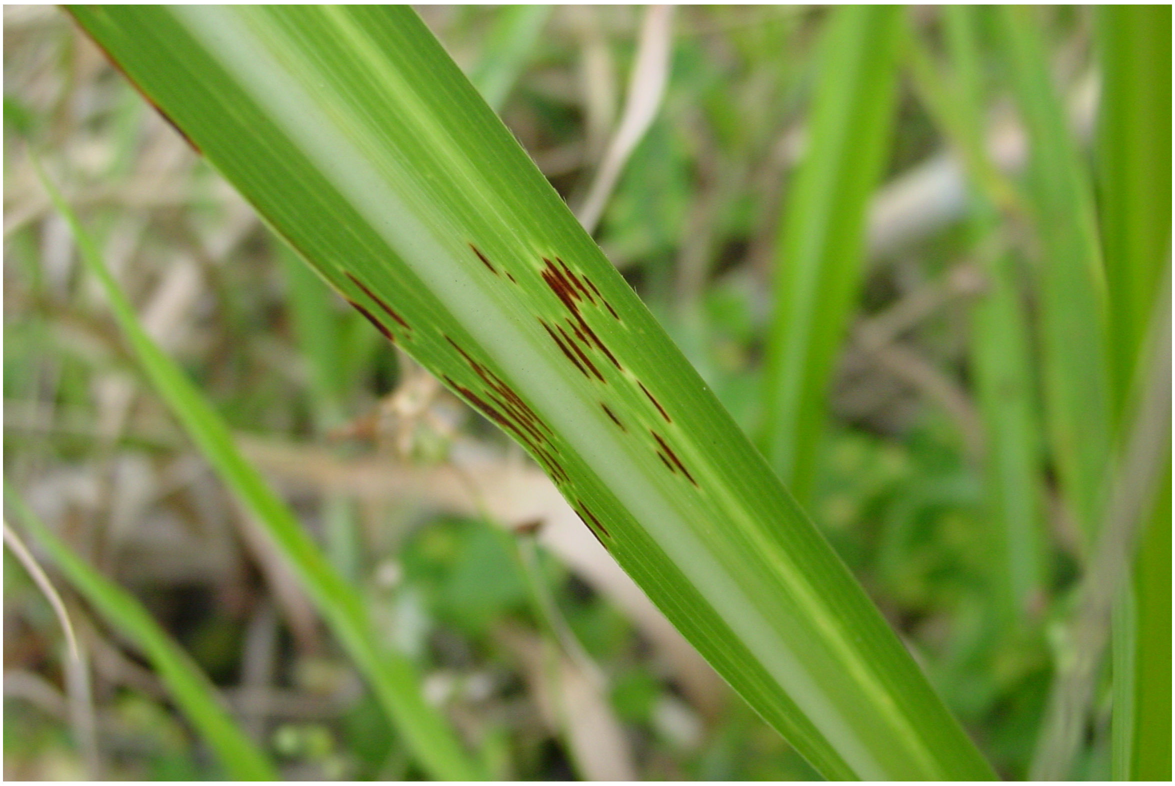

Fig. S1. Symptoms of red stripe disease formed on sugarcane leaves.

The disease symptoms were caused by natural infections in Okinawa Prefecture, Japan, in 2001 [10].

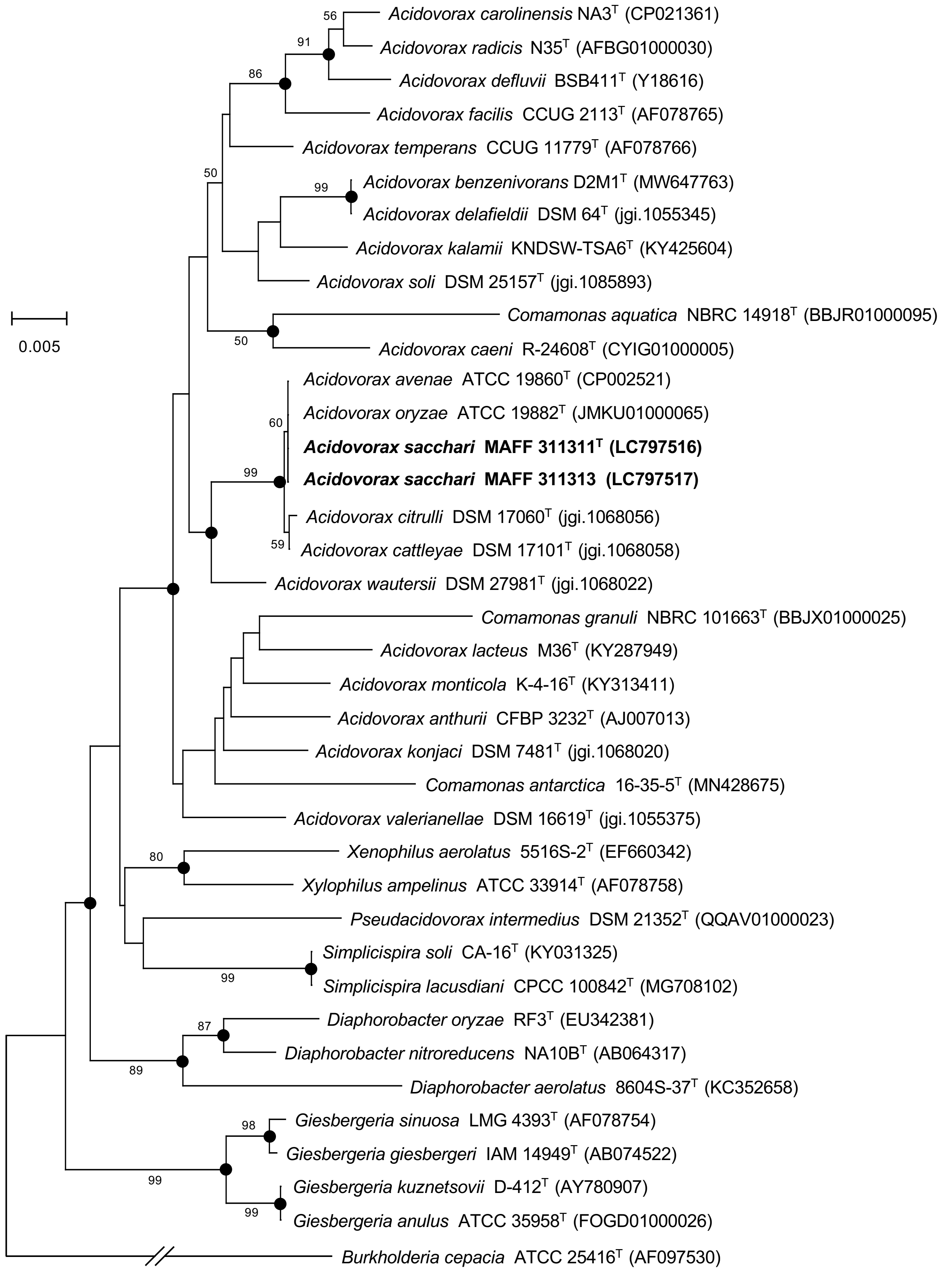

Fig. S2. Neighbour-joining tree based on the 16S rRNA gene sequences shows the relationships between *Acidovorax sacchari* sp. nov. strains (boldface type) and the closely related species (Table S4). *Burkholderia cepacia* ATCC 25416^{T} was used as an outgroup. Evolutionary distances were computed using the maximum composite likelihood method. Rate variation among sites was modelled with a gamma distribution (shape parameter = 1). T, type strain of the species. Numbers at nodes indicate the standard bootstrap values (≥ 50%) from 1000 repetitions. Filled circles indicate that the corresponding nodes were also recovered in the maximum-likelihood and maximum-parsimony trees. Bar, 0.005 substitutions per nucleotide position.

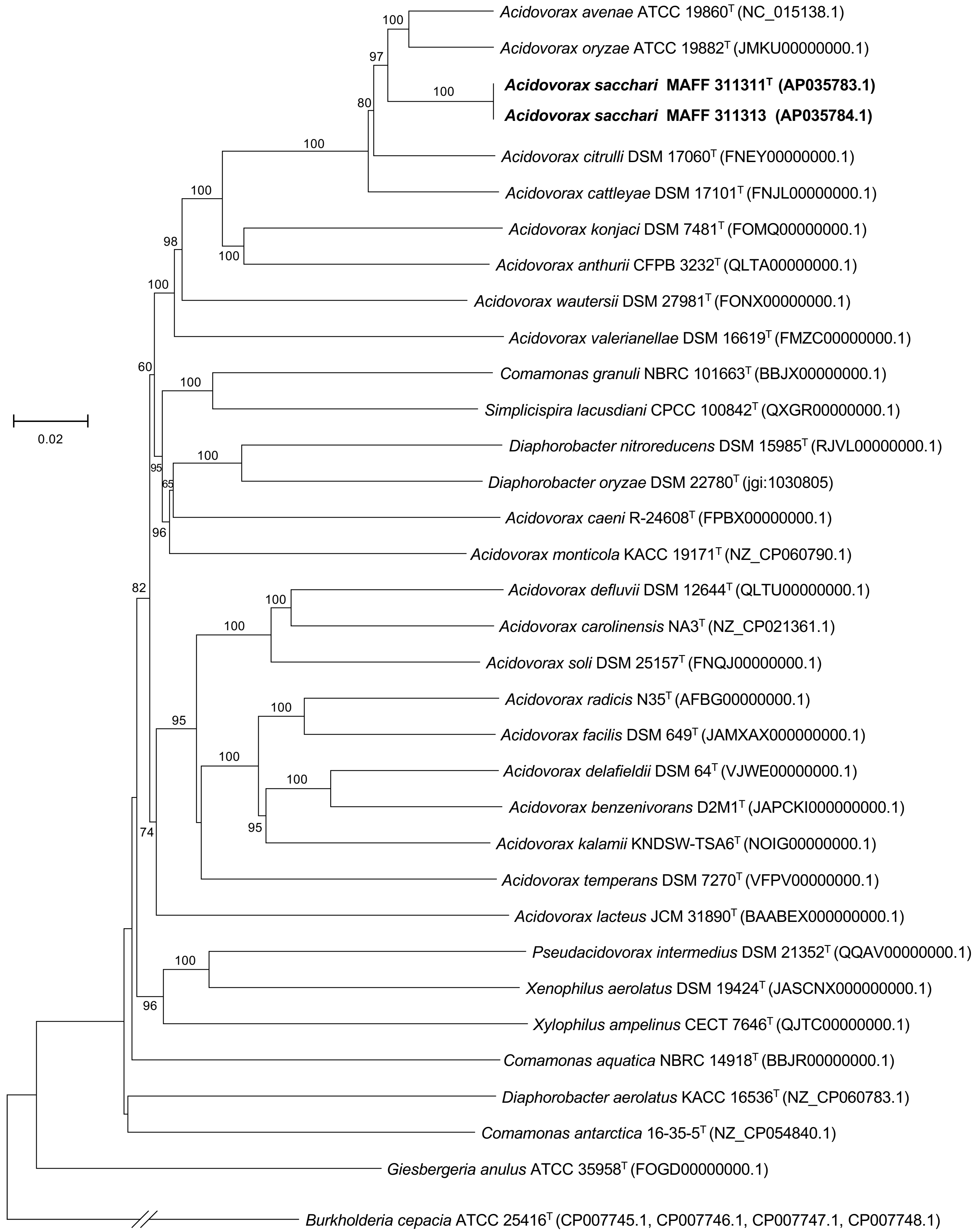

Fig. S3. Phylogenomic tree generated with FastME 2.1.6.1 from GBDP distances calculated from the genome sequences on the TYGS web server [18], showing the relationships between *Acidovorax sacchari* sp. nov. strains (boldface type) and the closely related species listed in Table 1. Branch lengths were scaled in terms of the GBDP distance formula d_5. *Burkholderia cepacia* ATCC 25416^T was used as an outgroup. GenBank accession numbers are shown in parentheses. T, type strain of the species. Numbers at nodes indicate the GBDP pseudo-bootstrap support values (≥ 60%) from 100 repetitions, with an average branch support of 87.1%.

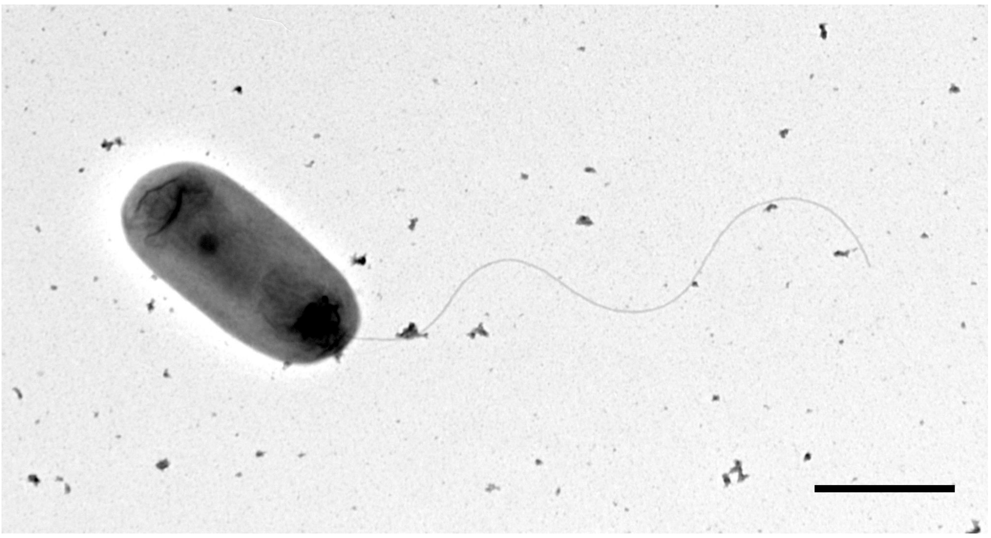

Fig. S4. Transmission electron micrograph of *Acidovorax sacchari* sp. nov. strain MAFF 311311^T, showing a rod-shaped cell and one polar flagellum. Scale bar, 1 μm.

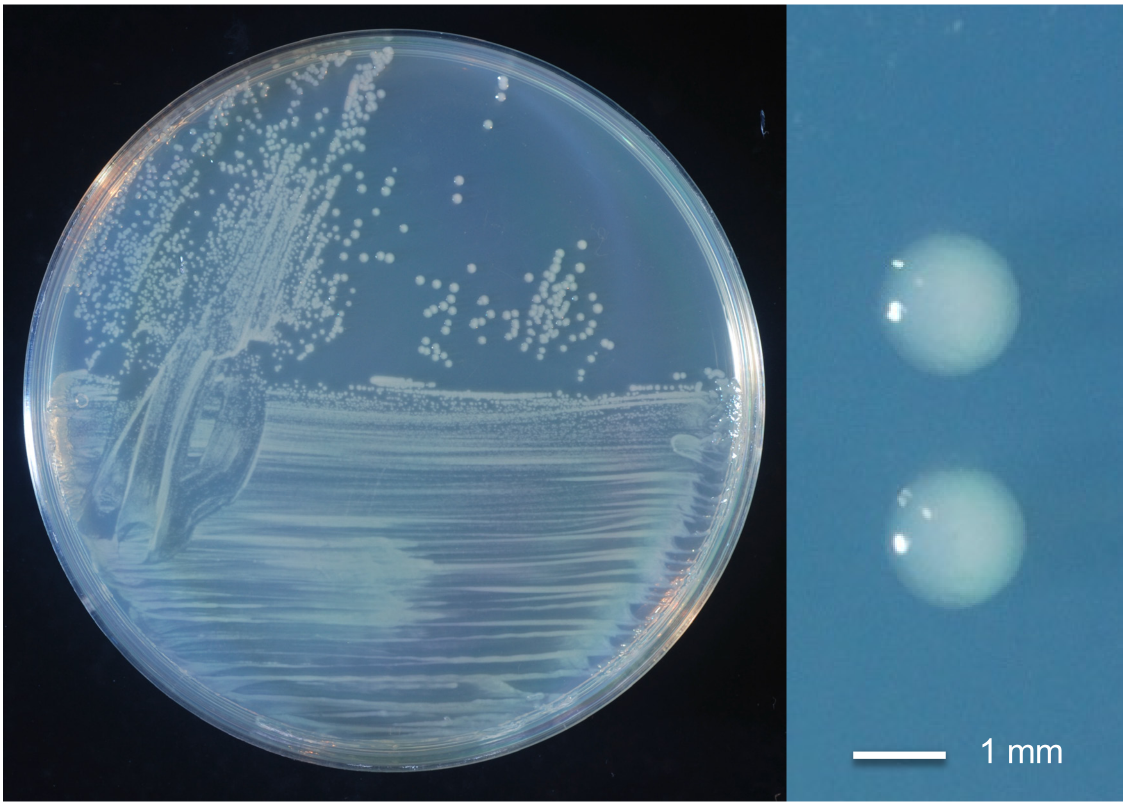

Fig. S5. Colony colour and morphology of *Acidovorax sacchari* sp. nov. strain MAFF 311311^T on a standard methods agar (plate count agar) plate incubated at 28 °C for 48 h.

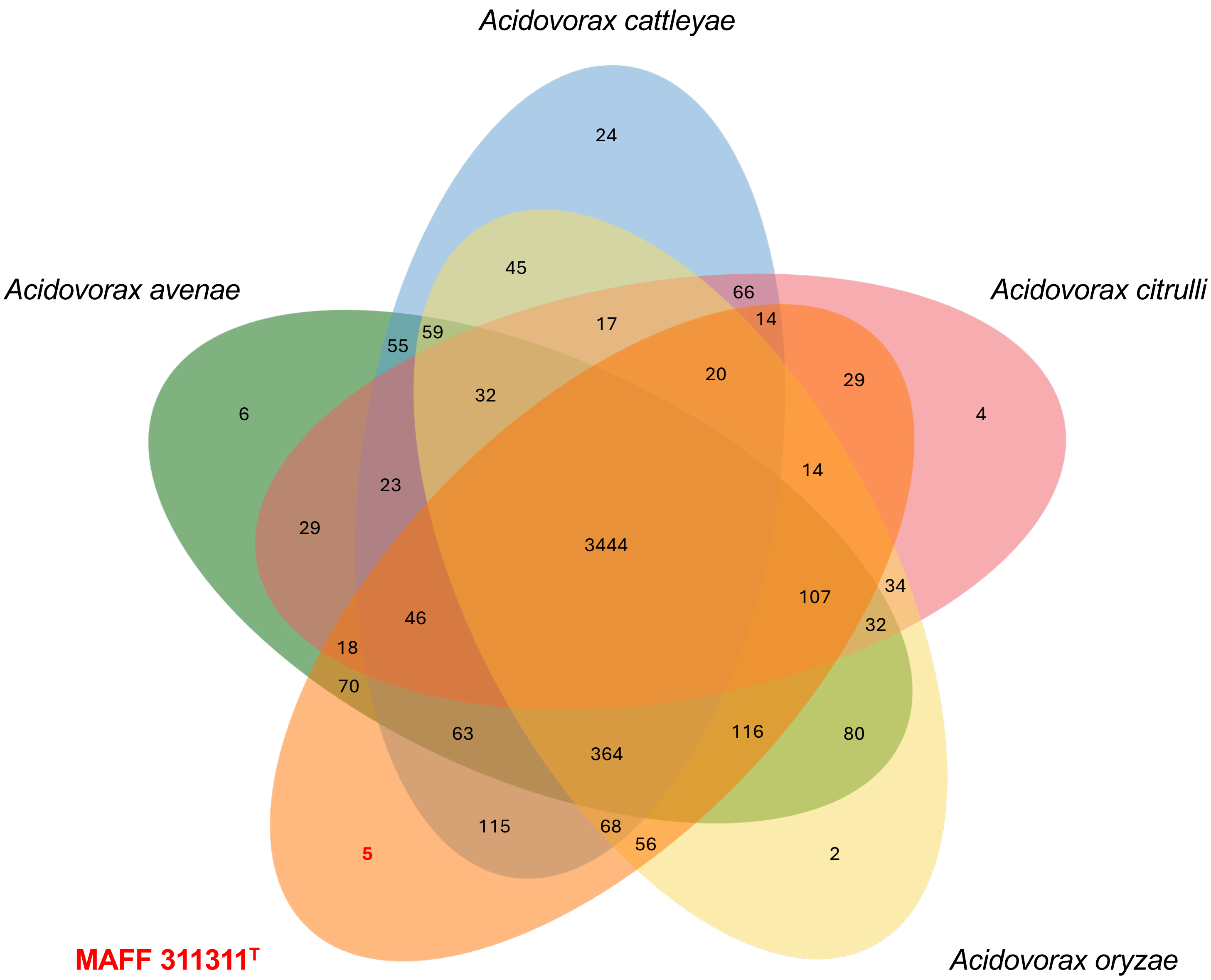

Fig. S6. Venn diagram, showing the orthologous gene clusters among *Acidovorax sacchari* MAFF 311311^T and its closely related species, generated using the OrthoVenn3 web server [46].

Table S1. Characteristics of the genome sequences

Characteristic	MAFF 311311^T	MAFF 311313
Total no. of contigs:		
Chromosome	1	1
Plasmid	0	0
Circular	Yes	Yes
DDBJ/ENA/GenBank Nucleotide accession number	AP035783	AP035784
Sequence Read Archive accession number	DRR576325	DRR576326
Size (bp)	5,583,543	5,583,543
DNA G+C content (mol%)	69.0	69.0
No. of CDSs	4,908	4,908
No. of rRNA genes	3, 3, 3 (5S, 16S, 23S)	3, 3, 3 (5S, 16S, 23S)
No. of tRNA genes	55	55
No. of CRISPRs	3	3
Coding ratio (%)	91.2	91.2
Completeness (%) *	99.83	99.83
Contamination (%) *	0.20	0.20

* The quality of the genome sequences was assessed using the CheckM [13].

Table S2. Pairwise comparisons of the MAFF 311311^T and type strain genomes using TYGS

TYGS (Type Strain Genome Server) [18] is available at https://tygs.dsmz.de. The data shown here were calculated with formula d_4 (formula d_4 is identical to formula 2 of GGDC 3.0) using the MAFF 311311^T genome sequence as a query and are presented in descending order of their dDDH values.

**Species**	**Strain**	**dDDH (d_4, in %)**	**Confidence intervals (d_4, in %)**
Acidovorax oryzae	ATCC 19882^T	54.3	[51.5 - 56.9]
Acidovorax avenae	ATCC 19860^T	52.6	[49.9 - 55.3]
Acidovorax citrulli	DSM 17060^T	50.9	[48.3 - 53.6]
Acidovorax cattleyae	DSM 17101^T	48.1	[45.5 - 50.7]
Acidovorax anthurii	DSM 16745^T	27.3	[25.0 - 29.8]
Acidovorax konjaci	DSM 7481^T	26.9	[24.6 - 29.4]
Acidovorax wautersii	DSM 27981^T	25.7	[23.4 - 28.2]
Acidovorax monticola	KACC 19171^T	23.7	[21.4 - 26.2]
Acidovorax valerianellae	DSM 16619^T	23.4	[21.1 - 25.9]
Diaphorobacter oryzae	DSM 22780^T	23.0	[20.7 - 25.5]
Acidovorax soli	DSM 25157^T	22.6	[20.3 - 25.1]
Acidovorax radicis	N35^T	22.4	[20.1 - 24.8]
Acidovorax temperans	DSM 7270^T	22.2	[19.9 - 24.6]

Table S3. Pairwise comparisons of the MAFF 311311^T and type strain genomes based on Taxonomy Check implemented in DFAST

DFAST (DDBJ Fast Annotation and Submission Tool) [14] is available at https://dfast.ddbj.nig.ac.jp. The data shown here were calculated with the FastANI algorithm [19] using the MAFF 311311^T genome sequence as a query and are presented in descending order of their ANI values.

Species	Strain	Accession	Taxonomy ID	ANI (%)	Matched fragments	Total fragments	Status
Acidovorax oryzae	ATCC 19882^T	GCA_000687165.1	862720	94.264	1562	1861	below_threshold
Acidovorax avenae subsp. *avenae*	ATCC 19860^T	GCA_000176855.2	80870	93.985	1621	1861	below_threshold
Acidovorax citrulli	DSM 17060^T	GCA_900100305.1	80869	93.632	1400	1861	below_threshold
Acidovorax cattleyae	DSM 17101^T	GCA_900104515.1	80868	92.978	1559	1861	below_threshold
Acidovorax anthurii	CFPB 3232^T	GCA_003269065.1	78229	85.782	1219	1861	below_threshold
Acidovorax konjaci	DSM 7481^T	GCA_900112675.1	32040	85.764	1280	1861	below_threshold
Acidovorax radicis	N35^T	GCA_000204195.2	758826	81.012	859	1861	below_threshold
Xylophilus ampelinus	CFBP 1192^T	GCA_024832295.1	54067	80.765	649	1861	below_threshold
Acidovorax facilis	DSM 649^T	GCA_023913775.1	12917	80.753	824	1861	below_threshold
Hydrogenophaga crocea	BA0156^T	GCA_011388215.1	2716225	80.288	719	1861	below_threshold
Diaphorobacter caeni	NR2-3-3-1^T	GCA_015354245.1	2784387	80.209	720	1861	below_threshold
Diaphorobacter aerolatus	KACC 16536^T	GCA_014489535.1	1288495	79.497	649	1861	below_threshold

Table S4. 16S rRNA gene sequence similarities between *Acidovorax sacchari* sp. nov. strain MAFF 311311^T and the type strains of the closely related species

Similarity values based on the 16S rRNA gene sequences were calculated using the pairwise nucleotide sequence alignment tool in EzBioCloud [22] with the MAFF 311311^T sequence as a query. The data are presented in descending order of the calculated similarity values.

Species	Strain	Accession number *	Pairwise similarity (%)
Acidovorax oryzae	ATCC 19882^T	JMKU01000065	100.00
Acidovorax avenae	ATCC 19860^T	CP002521	99.93
Acidovorax cattleyae	DSM 17101^T	jgi.1068058	99.93
Acidovorax citrulli	DSM 17060^T	jgi.1068056	99.86
Acidovorax wautersii	DSM 27981^T	jgi.1068022	98.90
Acidovorax konjaci	DSM 7481^T	jgi.1068020	98.41
Acidovorax temperans	CCUG 11779^T	AF078766	98.21
Acidovorax soli	DSM 25157^T	jgi.1085893	98.14
Acidovorax valerianellae	DSM 16619^T	jgi.1055375	98.00
Acidovorax anthurii	CFBP 3232^T	AJ007013	97.72
Acidovorax facilis	CCUG 2113^T	AF078765	97.66
Acidovorax kalamii	KNDSW-TSA6^T	KY425604	97.38
Acidovorax monticola	K-4-16^T	KY313411	97.37
Acidovorax delafieldii	DSM 64^T	jgi.1055345	97.31
Acidovorax caeni	R-24608^T	CYIG01000005	97.31
Acidovorax benzenivorans	D2M1^T	MW647763	97.30
Acidovorax defluvii	BSB411^T	Y18616	97.11
Pseudacidovorax intermedius	DSM 21352^T	QQAV01000023	97.11
Xylophilus ampelinus	ATCC 33914^T	AF078758	96.97
Acidovorax radicis	N35^T	AFBG01000030	96.97
Diaphorobacter nitroreducens	NA10B^T	AB064317	96.90
Acidovorax carolinensis	NA3^T	CP021361	96.90
Acidovorax lacteus	M36^T	KY287949	96.84
Giesbergeria giesbergeri	IAM 14949^T	AB074522	96.62
Giesbergeria anulus	ATCC 35958^T	FOGD01000026	96.55
Giesbergeria kuznetsovii	D-412^T	AY780907	96.52
Simplicispira lacusdiani	CPCC 100842^T	MG708102	96.49
Xenophilus aerolatus	5516S-2^T	EF660342	96.44
Comamonas aquatica	NBRC 14918^T	BBJR01000095	96.42
Giesbergeria sinuosa	LMG 4393^T	AF078754	96.42
Comamonas antarctica	16-35-5^T	MN428675	96.40
Comamonas granuli	NBRC 101663^T	BBJX01000025	96.35
Simplicispira soli	CA-16^T	KY031325	96.34
Diaphorobacter oryzae	RF3^T	EU342381	95.65
Diaphorobacter aerolatus	8604S-37^T	KC352658	94.94

* The 16S rRNA gene sequences used here are the same as those used in the phylogenetic analyses (Fig. S2).

Table S5. Number of genes in the MAFF 311311^{T} genome assigned to each COG functional category by using the eggNOG-Mapper *

Code	Description	Gene counts
**Information storage and processing**		
J	Translation, ribosomal structure and biogenesis	212
A	RNA processing and modification	1
K	Transcription	434
L	Replication, recombination and repair	156
B	Chromatin structure and dynamics	5
**Cellular processes and signaling**		
D	Cell cycle control, cell division, chromosome partitioning	48
V	Defense mechanisms	56
T	Signal transduction mechanisms	309
M	Cell wall/membrane/envelope biogenesis	280
N	Cell motility	164
U	Intracellular trafficking, secretion, and vesicular transport	124
O	Post-translational modification, protein turnover, chaperones	133
Z	Cytoskeleton	3
**Metabolism**		
C	Energy production and conversion	314
G	Carbohydrate transport and metabolism	236
E	Amino acid transport and metabolism	424
F	Nucleotide transport and metabolism	116
H	Coenzyme transport and metabolism	193
I	Lipid transport and metabolism	203
P	Inorganic ion transport and metabolism	334
Q	Secondary metabolites biosynthesis, transport and catabolism	158
**Poorly characterized**		
S	Function unknown	866
-	Not in COGs	251

* EggNOG-mapper online tool [37] is available at http://eggnog-mapper.embl.de.

Table S6. Number of genes in the genomes of MAFF 311311^T and its closely related *Acidovorax* species, assigned to the core components of each secretion system by using the BlastKOALA tool *

Strains (accession numbers): 1, *Acidovorax sacchari* MAFF 311311^T (AP035783.1); 2, *Acidovorax avenae* ATCC 19860^T (CP002521.1); 3, *Acidovorax oryzae* ATCC 19882^T (NZ_JMKU00000000.1); 4, *Acidovorax cattleyae* DSM 17101^T (FNJL00000000.1); 5, *Acidovorax citrulli* DSM 17060^T (NZ_FNEY00000000.1); 6, *Acidovorax facilis* DSM 649^T (JAMXAX000000000.1); 7, *Acidovorax delafieldii* DSM 64^T (VJWE00000000.1).

Secretion system	Number of genes assigned to each secretion system †						
	**1**	**2**	**3**	**4**	**5**	**6**	**7**
Type II secretion system	18	18	20	18	18	17	15
Sec secretory pathway	11	11	11	11	11	11	11
Tat secretory pathway	3	3	3	3	3	3	3
Type III secretion system ‡	10	10	10	10	10	0	0
Type VI secretion system	10	13	11	8	6	0	0

* BlastKOALA [38] is available at https://www.kegg.jp/blastkoala/.

† The total number of genes, assigned to the core components of each secretion system by using the BlastKOALA, is shown for each strain.

‡ Excluding the flagellar secretion system.

Table S7. Putative type III secreted effectors specific to MAFF 311311^T predicted by OrthoVenn3 and Bastion3 *

CDS information			Prediction results based on single models ‡										Prediction results based on final ensemble model ‡		
**Locus tag**	**Location †**	**Product †**	**AAC**	**DPC**	**QSO**	**CTDC**	**CTDT**	**PSSM-composition**	**RPM-PSSM**	**D-FPSSM**	**TPC**	**DP-PSSM**	**Score**	**T3SE**	**Type**
AVXHC19_22760	2525526.. 2526347	IclR family transcriptional regulator	0.961	0.944	0.981	0.981	0.925	0.000	0.967	0.955	0.013	0.000	0.672	Yes	Pred.
AVXHC19_18420	2039962.. 2040291	hypothetical protein	0.326	0.427	0.262	0.251	0.659	1.000	0.802	0.473	0.510	0.971	0.574	Yes	Pred.

* Effector predictions were made using Bastion3 [47] for the OrthoVenn3 results shown in Fig. S6.

† These data are based on the annotation results via DFAST [14].

‡ For detailed information on the prediction models, see the original publication of Bastion3 [47].

Table S8. Putative type VI secreted effectors specific to MAFF 311311^T predicted by OrthoVenn3 and Bastion6 *

CDS information			Prediction results based on single models ‡									Prediction results based on final ensemble model ‡		
**Locus tag**	**Location †**	**Product †**	**AAC**	**DPC**	**QSO**	**BLOSUM**	**DPC-PSSM**	**S-FPSSM**	**Pse-PSSM**	**CTDC**	**CTDT**	**Score**	**T6SE**	**Type**
AVXHC19_18410	2039581..2039904	hypothetical protein	0.848	0.773	0.852	0.718	0.921	0.780	0.917	0.949	0.760	0.838	Yes	Pred.
AVXHC19_18420	2039962..2040291	hypothetical protein	0.734	0.777	0.805	0.756	0.940	0.872	0.959	0.728	0.854	0.815	Yes	Pred.
AVXHC19_40330	4596394..4597446	phosphate ABC transporter substrate-binding protein PstS family protein	0.688	0.497	0.452	0.640	0.286	0.354	0.435	0.662	0.818	0.572	Yes	Pred.
AVXHC19_40340	4597443..4598480	phosphate ABC transporter substrate-binding protein	0.515	0.774	0.504	0.787	0.274	0.167	0.347	0.613	0.492	0.515	Yes	Pred.

* Effector predictions were made using Bastion6 [48] for the OrthoVenn3 results shown in Fig. S6.

† These data are based on the annotation results via DFAST [14].

‡ For detailed information on the prediction models, see the original publication of Bastion6 [48].
